# Lung cancer palindromia due to the deficiency of vital energy and blood syndrome treated by TCM herbs: A case report

**DOI:** 10.1097/MD.0000000000041203

**Published:** 2025-01-03

**Authors:** Xiaomeng Sun, Dongyang Tan, Jiarui Miao, Chang Wang, Yi Xu, Fei Yuan, Xu Fan

**Affiliations:** a Liaoning University of Traditional Chinese Medicine, Shenyang, China.

**Keywords:** case report, lung cancer therapy, lung malignancy, traditional Chinese medicine decoction

## Abstract

**Rationale:**

Lung cancer is a common primary malignant tumor of lung in clinic, which leads the world in both new cancers and new cancer deaths. At present, surgical treatment, radiotherapy and chemotherapy are commonly used in the treatment of lung cancer. However, due to the strong occultness of the diseases and the possibility of recurrence, the best treatment opportunity is often missed. Immunotherapy, which is the main method of lung cancer, but the treatment efficiency and survival are not satisfactory, most patients will have local recurrence or distant metastasis. Traditional Chinese medicine has a targeted effect on the treatment of lung cancer with small adverse reactions, and has a positive impact on alleviating the adverse reactions of radiotherapy and chemotherapy and prolonging the life span of patients. More and more attention should be paid to the research of lung cancer treatments.

**Patient concerns:**

Patients with lung tumors, which had been surgically removed in the past. This time, the tumor occurred again, and the patient could not tolerate another surgical treatment. Other targeted treatments are not available due to financial and body constraints.

**Diagnoses:**

The case was diagnosed as invasive adenocarcinoma of the upper lobe of the right lung.

**Interventions:**

The patient received early surgical treatment, and then changed to traditional Chinese medicine (TCM) decoction treatment. The treatment principle of Chinese medicine is mainly to tonify healthy *Qi* and blood, enhancing the immunity of the body.

**Outcomes:**

After nearly 2 years of TCM decoction treatment, the recurrent tumor has shrunk significantly. No discomfort such as fatigue, fever or weight loss. During this period, the patient did not receive western medical treatment such as surgery and chemotherapy, which did not affect normal life. It also proves that Chinese medicine is effective.

**Lessons:**

This case report confirms that traditional Chinese medicine is safe and effective in the treatment of lung cancer, improving the ability of strengthening the health, driving away evil, and fighting cancer. TCM herbs can block the lesion of new blood vessels, anticancer metastasis, prevent recurrence after surgery, which is worth promoting. In clinical practice, the individual treatment of patients and the symptomatic treatment according to different syndromes should be attached great importance.

## 1. Introduction

Lung cancer leads the world in both new cancers and new cancer deaths. The incidence and mortality rate of lung cancer in China is very high, which seriously affects the quality of life of patients. Affected by population aging, air pollution and smoking, the incidence and mortality of lung cancer are increasing year by year. China has become the country with the largest number of lung cancer patients. It is predicted that the number of lung cancer patients in China will reach 1 million by 2025.^[1,2]^

At present, surgical treatment, radiotherapy, and chemotherapy are commonly used to treat lung cancer. However, due to the strong occultness of lung cancer, the best treatment opportunity is often missed when it is diagnosed. In recent years, immunosuppressors have been used in the treatment of various cancers, but the proportion of people benefiting from them is still not high.^[3]^ Surgery, radiotherapy, chemotherapy, and other means for lung cancer patients are also very damaging.^[4]^

In recent years, Chinese medicine treatment of lung cancer has become a hot spot. More and more attention has been paid to the research of lung cancer. TCM treatment of lung cancer emphasizes the treatment of syndrome differentiation, the treatment of both symptoms and root causes, which makes Chinese medicine have advantages in the treatment of lung tumor. According to traditional Chinese medicine, lung cancer is caused by the insinuation of toxic pathogens due to the lack of healthy *Qi*, the mixture of deficiency and demonstration. Traditional Chinese medicine decoction has the effect of helping weak and increasing healthy *Qi*, which is widely recognized in clinical practice. Therefore, a case report is shared here for the benefit of readers. It makes the clinical application of TCM more standardized and accurate, and provides more choices for the clinical treatment of lung cancer.

## 2. Case report

### 2.1. Clinical information

**Figure 1.**
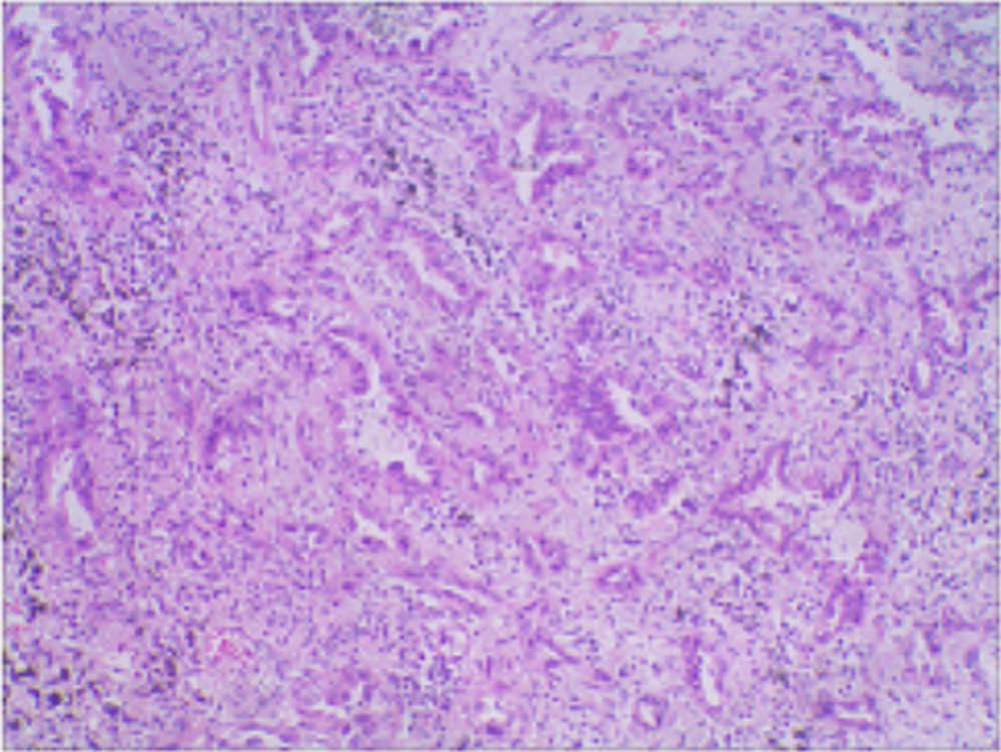
Pathological findings showed that there was an invasive adenocarcinoma in the upper lobe of the right lung with a size of 1.3 * 1.2 * 1 cm, involving the pleura, and no obvious neurovascular invasion.

The patient, female, 57 years old. In September 2021, lung tumors were found during the examination in The First Affiliated Hospital of Dalian Medical University. The chief complaint was chest pain and fatigue for half a year. For further treatment, the outpatient department was admitted to the hospital on the grounds of “lung shadow.” The patient’s mental state is good, appetite is reasonable, sleep is good, and weight has no significant change. Based on MSCT plain scan of the chest, partial solid nodules appeared in the posterior segment of the upper lobe of the patient’s right lung (21 mm * 13 mm), with signs of lobulation, burrs, pleural indentation, and vacuole. Multiple small nodules in both lungs. Blood test: neutrophils↑, lymphocytes↓. The patient underwent surgical treatment and part of lung tissue was removed. Pathology revealed invasive adenocarcinoma in the upper lobe of the right lung, and no metastasis in the parbronchial lymph nodes (Fig. 1).

### 2.2. TCM therapy

In November 2022, when the patient returned to the doctor, it was found that there were ground glass nodules in the upper lobe of the left lung, about 18 mm * 9 mm, indicating the recurrence of the primary cancer. The patient refused a second surgical treatment. The symptoms were fatigue, pale tongue, white fur, and weak pulse. TCM syndrome is *Qi* and blood deficiency, the treatment principle is tonifying *Qi* and blood, attacking evil, and removing blood stasis. The Chinese medicine prescription is as follows: Shashen (*Adenophora stricta*) 20 g, Baihe (*Lilium brownii*) 20 g, Dangshen (*Codonopsis pilosula*) 20 g, Chuanbeimu (*Sichuan fritillary bulb*) 15 g, Baihuasheshecao (*Hedyotis diffusa Willd*) 15 g, Jiegeng (*Platycodon grandiflorus*) 20 g, Maidong (*Ophiopogon japonicus*) 20 g, Sangbaipi (*Mulberry root bark*) 15 g, Shudi (*Rehmannia glutinosa*) 10 g, Danggui (*Chinese angelica*) 8 g, Fuling (*Poria cocos*) 20 g, Guiban (tortoiseshell) 8 g, Biejia (*Carapax trionycis*) 8 g, Gouqizi (*Lycium chinense*) 15 g, Niuxi (*radix achyranthis bidentatae*) 15 g, Muli (*Concha Ostreae*) 20 g. One dose per day, totaling 180 mL, twice a day in the morning and evening, half an hour after meals, lasting 1.5 months.

### 2.3. Treatment outcomes

After that, the patient took Chinese medicine and had a semiannual physical examination. In January 2024, The CT examination revealed a recurrent tumor shrinkage of about 7 × 9 mm, which was undoubtedly amazing. Blood routine examination was normal, liver and kidney function was normal. The patient had no signs of fatigue or wasting, and diet, bowel movements and sleep were normal. It shows that the treatment plan of traditional Chinese medicine is correct and the medication is symptomatic.

**Figure 2.**
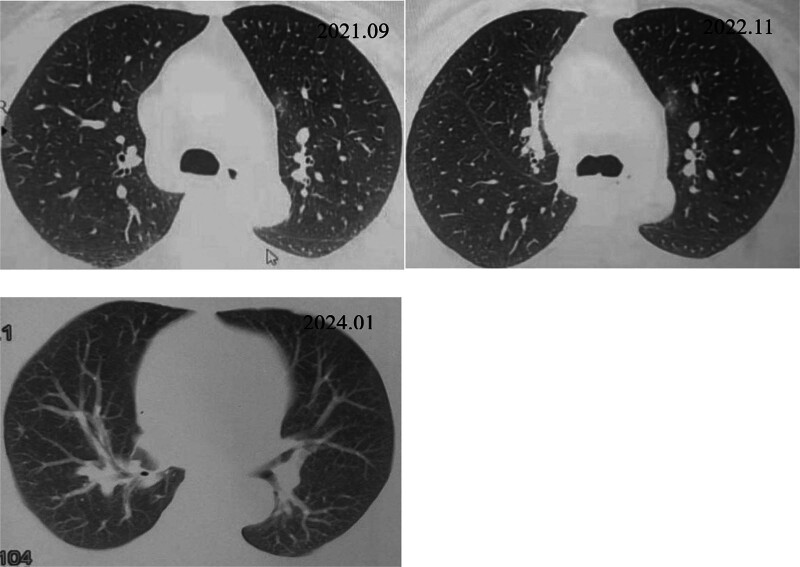
The CT images show changes in lungs before and after treatment.

From 2021 to 2024, the patient underwent a follow-up examination of the lung tissue, and CT showed that the size of the lung masses changed as shown in Table 1 and Figure 2, indicating that the lung tumors became significantly smaller during treatment after lung CT examination. The level of carcino-embryonic antigen tends to be normal. For this patient, the lung cancer recurred after surgery and could not tolerate another operation, so she sought Chinese medicine treatment. And achieved the expected desired efficacy. As shown in Figure 2, before TCM treatment, the CT showed that dense shadows and cord foci could be seen in the hilar area of the lung. Ground glass nodules were observed in the upper lobe of the left lung. There are small nodules in both lungs and multiple small lymph nodes in the mediastinum. After treatment, the image showed that the lung texture became clearer and the nodules shrinked. Calcification shadow and cord foci lessened or even disappeared. The author believes that this treatment plan is worth promoting.

**Table 1:** Multislice spiral CT results of the chest.

Examination date	Conclusion of CT	Size
September 14, 2021	Nodules were observed in the posterior upper lobe of the right lung, considering the possibility of malignancy	Maximum approximately 21 * 13 mm
November 18, 2022	The volume of the right lung was reduced, and the upper lobe of the left lung showed frosted glassy nodule, considered primary cancer, small nodules of both lungs	Maximum approximately 18 * 9 mm
January 5, 2024	The volume of the right lung was reduced and the nodules of the upper lobe of the left lung were smaller than before	Maximum approximately 7 * 9 mm

## 3. Discussion

The incidence of lung cancer is severe, the mortality is high, the prognosis is poor, and should be paid high attention. Traditional Chinese medicine has remarkable clinical efficacy and unique therapeutic advantages in the treatment of lung cancer. The combination of traditional Chinese and western medicine is expected to become the mainstream trend of cancer treatment today. The holistic concept of traditional Chinese medicine should be brought into full play by multi-dimensional combination.^[4]^ Compared with traditional Chinese medicine monomer, decoction of medicinal herbs have the characteristics of multi-component, low drug resistance and low adverse reactions, and has become an effective means to treat lung cancer.

According to traditional Chinese medicine, the main etiology and pathogenesis of lung cancer include deficiency of healthy *Qi*, deficiency of *Qi* and *Yin*, invasion of lungs by toxic substance, internal accumulation of phlegm and blood stasis, resulting in loss of lung circulation, retention of evil *Qi* in the lungs, depletion of *Qi* and *Yin*, and lung tumor over time.^[5]^ The disease is located in the lungs. Essence is the loss of *Qi* and *Yin* 2 False. Supplementing deficiency is what traditional Chinese medicine is good at, so the principle of treatment should be the method of supplementing *Qi* and nourishing *Yin* and moistening lung.

As a traditional Chinese culture, TCM has a long history. Chinese medicine used in the treatment of malignant tumors can play a significant role in reducing toxicity, sensitizing, increasing curative effect, improving patients’ quality of life and improving prognosis. In the prescription, *C pilosula* supplied *Qi*-blood, *A stricta* and *O japonicus* nourishing *Yin* and moistening lung. Modern pharmacology has confirmed that the effective monomer components of Shashen (*Radix adenophora*) have the effects of regulating immune function, antitumor, anti-inflammation and anti-oxidation.^[6]^ Maidong (*O japonicus*) contains steroid saponins, high isoflavones, polysaccharides, volatile oil, amino acids, and trace elements, which can achieve the sensitized effect on lung cancer and other tumor cells, inhibit tumor growth and metastasis, and the therapeutic effect is exact.^[7]^ The composition and efficacy of turtle shell (*C trionycis*) have been studied most deeply in modern times. It is rich in proteins, amino acids, peptides, fatty acids, macroelements, trace elements and volatile components. Some studies have shown that the components with good antitumor activity were screened from the hydrolysates of alkali-soluble protein neutral protease of soft-shelled turtle, which had better inhibitory effect on human lung cancer A549 cell.^[8]^ The anticancer effect of *C angelica* is mainly reflected by ASP and Z-LIG, and its related mechanisms including anti-proliferation, induction of tumor cell apoptosis, anti-metastasis, inhibition of tumor angiogenesis, regulation of immunity and other molecular and signaling mechanisms.^[9,10]^ The steroids, flavonoids, and polysaccharides in *Mulberry root bark* have certain antioxidant capacity,^[11]^ and could inhibit a variety of tumor cells.^[12]^ It is a classic drug for releasing lung *Qi* and helping the elimination of toxicant.

When all medicines are used together, the combination of various drugs can increase the antitumor effect, restore the patient’s *Qi* and blood, improve the patient’s physique and improve the patient’s quality of life. Meanwhile, the meridian of the lung will also be dredged, and the antitumor mechanism will be used together.

## 4. Conclusion

As a traditional treatment method, TCM plays an increasingly important role in the prevention and treatment of lung cancer. Some traditional Chinese medicine and traditional Chinese medicine prescriptions have been proved to have antitumor activity and can regulate a variety of signaling pathways, thus affecting the invasion and metastasis of lung cancer cells. Its essence is to focus on the source, on the basis of strengthening and regulating the internal environment, to inhibit the development of tumors.^[13]^ It was found in this case that *Qi* deficiency and blood deficiency were the main syndrome types of this patient with lung adenocarcinoma.

However, due to the numerous pathological types of lung cancer itself and its rapid progress, how to better learn the effective experience of syndrome differentiation and treatment of lung cancer from the treasure house of traditional Chinese medicine in the perspective of modern medical research, and combine the relatively stable syndrome differentiation system with flexible individual particularity? Maybe as a preventive care after surgery, reduce the recurrence rate, improve the quality of life measures, so as to help clinical treatment, which need further in-depth research. In addition, as a single case, the limitation of this study is that there are data biases due to factors such as sample size, funds, and regional distribution of population. As well as the integrity of TCM syndrome type, it needs to be gradually improved in the future research to provide better reference for evidence-based medicine.

## Acknowledgments

The authors thank the participants and the numerous team members involved in the studies, including Xiaomeng Sun, Dongyang Tan, Xu Fan, Jiarui Miao, Chang Wang, Yi Xu, Fei Yuan. They also thank the funders for their financial input and support.

## Author contributions

**Investigation:** Yi Xu, Fei Yuan.

**Writing – original draft:** Xiaomeng Sun, Dongyang Tan, Jiarui Miao, Chang Wang.

**Writing – review & editing:** Xu Fan.

## References

[1] Bray F Ferlay J Soerjomataram I Siegel RL Torre LA Jemal A. Global cancer statistics 2018: GLOBOCAN estimates of incidence and mortality worldwide for 36 cancers in 185 countries. CA Cancer J Clin. 2018;68:394–424. 30207593 10.3322/caac.21492

[2] Jin FG. The present situation of early screening of lung cancer in China. Med Philos. 2017;38:14–8.

[3] Tian JZ Wang HW. Study on the research model of TCM multi-dimensional combination drugs based on TCM prescription. J Shandong Univ TCM. 2011;35:99–101,120.

[4] Sui H Ma N Wang Y. Anti-PD-1/PD-L1 therapy for non-small-cell lung cancer: toward personalized medicine and combination strategies. J Immunol Res. 2018;8:6984948. 10.1155/2018/6984948 PMC6109480 30159341

[5] Qian WN Li Z Li RT. Clinical research on Qi-replenishing and Yin-nourishing prescription combined with TP chemotherapy in treatment of Qi-Yin deficiency of non-small cell lung cancer. Acta Chin Med. 2021;36:869–74.

[6] Yu L Zhou YP Zhou SH. Effects of Shashen Maidong Decoction on oxidative stress, inflammation and apoptosis of Lewis lung cancer model mice. Lishizhen Med Mater Med. 2023;34:317–20.

[7] Liu Q Lu JJ Hong HJ Yang Q Wang Y Chen X-J. *Ophiopogon japonicus* and its active compounds: a review of potential anticancer effects and underlying mechanisms. Phytomedicine. 2023;113:154718. 36854203 10.1016/j.phymed.2023.154718

[8] Wu Y Liu X Wang JL. Soft-shelled turtle peptide modulates microRNA profile in human gastric cancer AGS cells. Oncol Lett. 2018;15:3109–20. 29435044 10.3892/ol.2017.7692 PMC5778892

[9] Yang J Shao XY Jiang J. Angelica sinensis polysaccharide inhibits proliferation, migration, and invasion by downregulating microRNA-675 in human neuroblastoma cell line SH-SY5Y. Cell Biol Int. 2018;42:867–76. 29465760 10.1002/cbin.10954

[10] Chen L Huang GL. Antitumor activity of polysaccharides: an overview. Curr Drug Targets. 2018;19:89–96. 28676001 10.2174/1389450118666170704143018

[11] Ding QY Ma SC Xu FG. Research progress on chemical constituents, pharmacology, and quality control of Mori Cortex. Chin J Pharm Anal. 2021;41:1114–24.

[12] Park SH Chi GY Eom HS. Role of autophagy in apoptosis induction by methylene chloride extracts of Mori Cortex in NCI-H460 human lung carcinoma cells. Int J Oncol. 2012;40:1929–40. 22367066 10.3892/ijo.2012.1386

[13] Cheng QW Zhou HL Mao QY. LIN Hong-sheng’s rehabilitation concept of “Wuyang” for malignant tumor under the guidance of the theory of “consolidating body resistance and treating root cause”. China J Tradit Chin Med. 2020;35:6141–3.

